# Saliva Malondialdehyde Concentration of Dogs With and Without Periodontal Disease

**DOI:** 10.1177/08987564241248042

**Published:** 2024-05-01

**Authors:** M. Schroers, K. Reiser, T. Alexander, Y. Zablotski, A. Meyer-Lindenberg

**Affiliations:** 1 Veterinary Faculty, 9183 Ludwig-Maximilians-Universitat Munchen, Munchen, Germany; 2 Tierklinik Gessertshausen, Munich, Germany

**Keywords:** malondialdehyde, MDA, oxidative stress, diagnostics, saliva, periodontal disease, periodontitis, dogs

## Abstract

The study investigated whether malondialdehyde (MDA), a biomarker for oxidative stress, can be used as a viable parameter in dog saliva for the diagnosis or early detection of periodontal disease (PD). Saliva MDA concentrations were measured preoperatively in dogs diagnosed with PD during dental prophylaxis and compared with those of dentally healthy dogs. 35 dogs were included in the study. The average MDA concentration was 270 ng/ml (range 27-633) in the dogs without PD (n = 10) and 183 (36-833) ng/ml (ng/ml) in the dogs with PD (n = 25). The maximum MDA concentration in the study group (PD ≥1) was 833 ng/ml, which was significantly higher than in the study group (PD = 0) (p<0.05). The study showed that salivary MDA concentrations could not distinguish between healthy dogs and those with PD.

## Introduction

Within the field of small animal veterinary dentistry, few diseases are as prevalent as periodontal disease in dogs and cats.^1–5^ Periodontitis is considered an inflammatory disease of the periodontium caused by a biofilm of microbes and bacteria.^6–8^ It is assumed that it is not the bacterial colonies themselves, but an impaired immune response to these bacterial colonies that is responsible for inflammation and destruction of the periodontium.^9–12^ Thus, environmental and genetic factors, such as breed and age, favor the predisposition to the disease.^13^ Genetic defects, such as mutations in genes associated with elevated levels of interleukin-1 (IL-1), have been shown to enhance the inflammatory response to periodontopathogenic bacteria in dogs.^14^ As a result of the inflammatory reaction, there is a loss of collagen fibers and atrophy of the gingiva and the periodontal ligament as well as resorption of the alveolar bone.^15^ In turn, the cytokines stimulate catabolic processes in response to the inflammation causing collagen and bone degradation which is promoted by matrix metalloproteinase.^16^ Environmental conditions adjacent to the periodontal tissue favor facultative anaerobic bacteria resulting in an increase in gram-negative bacteria.^17^ The growing presence of gram-negative bacteria results in the release of neutrophils, enzymes and oxygen-reactive species.^18^ The pathophysiological mechanisms are therefore associated with the oxidative stress caused by a dysfunction of the mitochondria.^19^ At the same time, the amount of antioxidants in saliva also decreases, which correlates with a human clinical study.^20^

Malondialdehyde (MDA), a degradation product of lipid peroxidation, has been established as a promising biomarker for oxidative stress in both human and veterinary medicine.^21–24^ In veterinary medicine, a pathological study of periodontal tissue in 64 cats showed that lipid peroxidation in the tissue is increased in patients with gingivitis and periodontitis.^25^ Another study also showed that systemic MDA levels were higher in cats with periodontal disease (PD) than in the dentally healthy control group.^26^

In dogs, the biomarker has previously been used to measure oxidative stress in the blood of patients with heart defects, as well as in the feces of patients with parvoviral enteritis.^27,28^ To the authors’ knowledge, no studies on saliva MDA concentrations of dogs with PD have been published.

Dental prophylaxis is usually performed under anesthesia in dogs and cats, which poses additional risks, specifically as many of the patients are geriatric with comorbidities. Therefore, a noninvasive salivary biomarker for PD could be helpful as an indicator that further treatment under anesthesia is needed.

This prospective study was designed to measure preoperative saliva MDA concentrations in dogs undergoing dental prophylaxis. Dental radiographs were used for staging PD in the study group. The aim was to verify if there is a correlation between saliva MDA concentrations and stage of the PD in dogs.

## Materials and Methods

The study was approved by the Ethics Committee of the Faculty of Veterinary Medicine, Maximilian University, Munich (AZ 284-13-09-2021). In the present study, dogs were obtained from the Clinic for Small Animal Surgery and Reproduction (Ludwig-Maximilians-University Munich), the Veterinary Clinic Gessertshausen and the Veterinary Practice Hadern, Munich, Germany. There were 19 male and 16 female dogs, the mean weight was 17 kg (range 2-43). The mean age of the study and control groups was 9 years and 4 years, respectively. The mean age of both groups was 8 years. The youngest age was 7 months. Dogs that underwent dental prophylaxis including dental radiography were included in the study group. Three patients with persistent primary teeth, four patients with malocclusion, one patient with hyperdontia and one patient with epulis were included. Dogs with inflammatory diseases (e.g., periapical radiographic lucency, tooth fracture) or tumors were excluded. During the preanesthetic examination, the patient's medical history was obtained, a general examination was performed and a blood analysis (i.e., red blood cell count, white blood cell count, biochemistry) was obtained. Patients without known preexisting conditions and normal preanesthetic examination were included in the study.

A saliva sample was taken from all dogs to measure MDA. The dogs were not allowed to eat for at least two hours before the saliva sample was taken. The patients in the study group were fasted due to anesthesia requirements for dental treatment. Saliva was obtained using a swab^a^ placed in the mouth under the tongue for 1 min. The swabs were then carefully removed and stored in a designated tube^b^, centrifuged^c^ (3000 r/min for 10 min), and the saliva transferred to a microreaction vessel^d^ with the aid of a pipette^e^ and deep-frozen (−20 °C). The evaluation was performed using a commercial Vasopressin ELISA kit^f^ in the in-house laboratory. For this purpose, the ELISA plate was first washed twice, the samples were thawed and then diluted with an appropriate buffer solution and then pipetted onto the ELISA plate. The biotin-detection-antibody working solution was pipetted and the plate was incubated covered at 37 °C for 45 min. In the next step, the ELISA plate was washed 3 times, the HRP-streptavidin conjugate (SABC) solution was pipetted and the plate was incubated again at 37 °C for 30 min. After washing the plate 5 times, the TMB substrate, which was also prewarmed at 37 °C, was pipetted and the plate was covered again and incubated at 37 °C for 15 min. Finally, a stop solution was pipetted and the plate was immediately measured.^g^

The staging of PD in the present study was based on an established staging of PD by the American Veterinary Dentistry College (AVDC).^29^ However, this only refers to the respective affected tooth and is derived from the distance from the alveolar margin to the enamel–cemental junction relative to the root length on the basis of radiographs (Table 1).

**Table 1.** Stages of Periodontal Disease.^29^

Stage	Abbreviation	Description
Normal	PD 0	Healthy gingiva
1	PD 1	Gingivitis without gingival atrophy
2	PD 2	Early stage with <25% loss of periodontium
3	PD 3	Moderate periodontitis with 25-50% loss of periodontium
4	PD 4	Advanced periodontitis with more than 50% loss of periodontium

Abbreviation: PD, periodontal disease.

When several teeth were affected, the worst degree of PD was scored. The number of affected teeth was also recorded. As the present study was only a pilot study, we used both the staging of the AVDC and a separate scoring system to describe the extent of PD. The extent was determined by the number of quadrants affected and points were awarded accordingly for the statistical evaluation (Table 2). The staging was performed by an approved specialist for small animals with a main focus on veterinary dentistry.

**Table 2.** Extent of Periodontal Disease Based on the Number of Quadrants Affected.

Points	Teeth or quadrants affected
0	No teeth
1	1 tooth
2	Several teeth in 1 or 2 quadrants
3	Several teeth in 3 or 4 quadrants

In addition, incidental findings of the dental examination were recorded in the diseased dogs (e.g., fourth premolar periapical lucency, tooth fracture, tumor). Patients with secondary findings that pointed to an inflammatory disease or tumor were subsequently excluded.

The clinical phase of the study was followed by the statistical evaluation. The results of the MDA measurements and the degree of PD were evaluated using the Kruskal–Wallis and Mann–Whitney U tests.

## Results

In total, saliva MDA concentrations were measured in 35 dogs, including 25 dogs with PD (study group) and 10 dogs without PD (control group).

**Figure 1.**
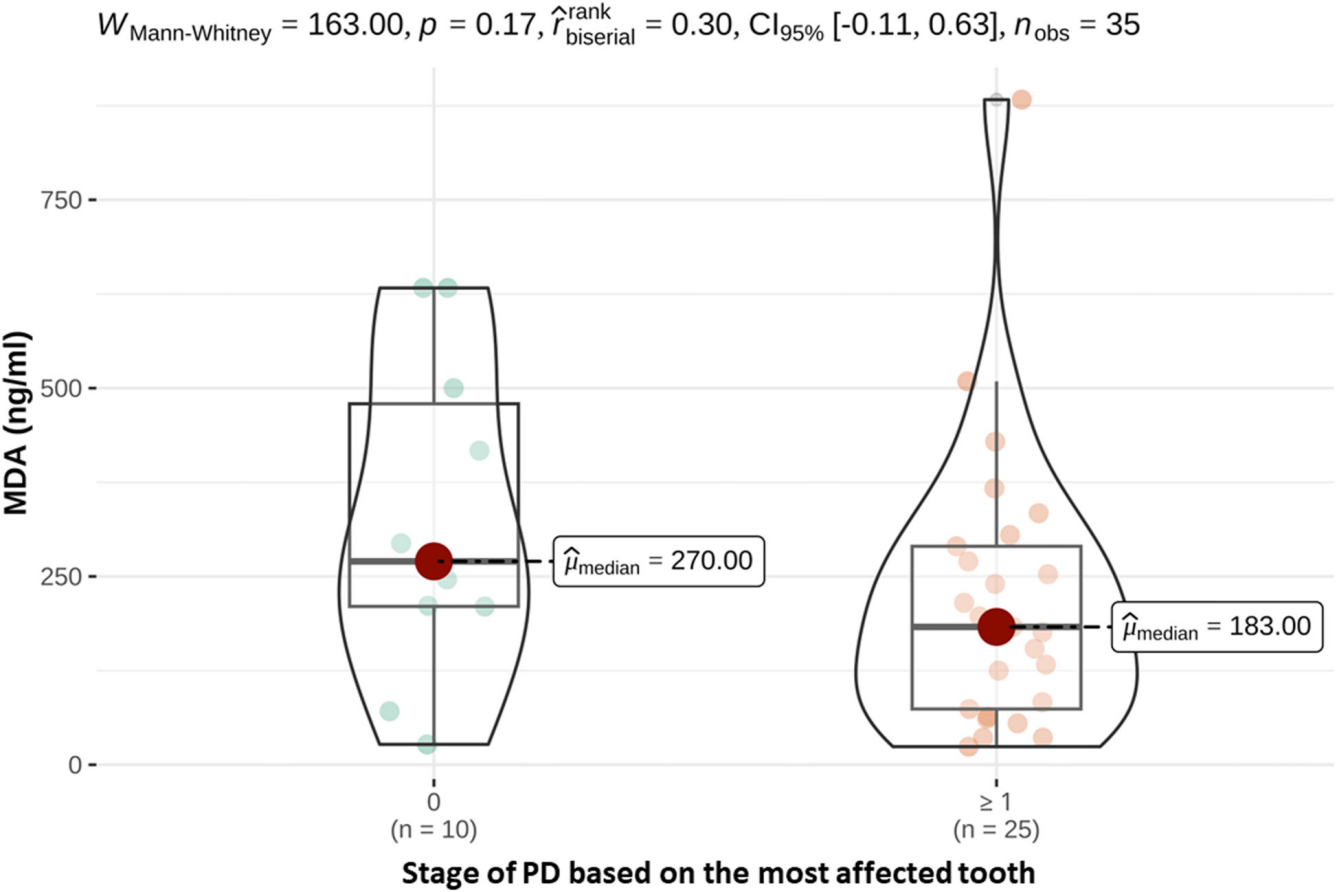
Saliva MDA concentrations (ng/ml) of healthy dogs (PD 0) and dogs suffering from periodontal disease (PD ≥ 1).

Abbreviations: MDA, malondialdehyde; PD, periodontal disease.

Saliva MDA concentrations were measured in all dogs. The mean MDA concentration was 183 ng/ml (range 36-833) in the study group and 270 ng/ml (range 27-633) in the control group. However, no significant differences were seen overall between the study and control groups (*p* = .17). The maximum MDA concentration in the study group (PD ≥1) was 833 ng/ml, which was significantly higher than in the study group (PD = 0) (Figure 1).

**Figure 2.**
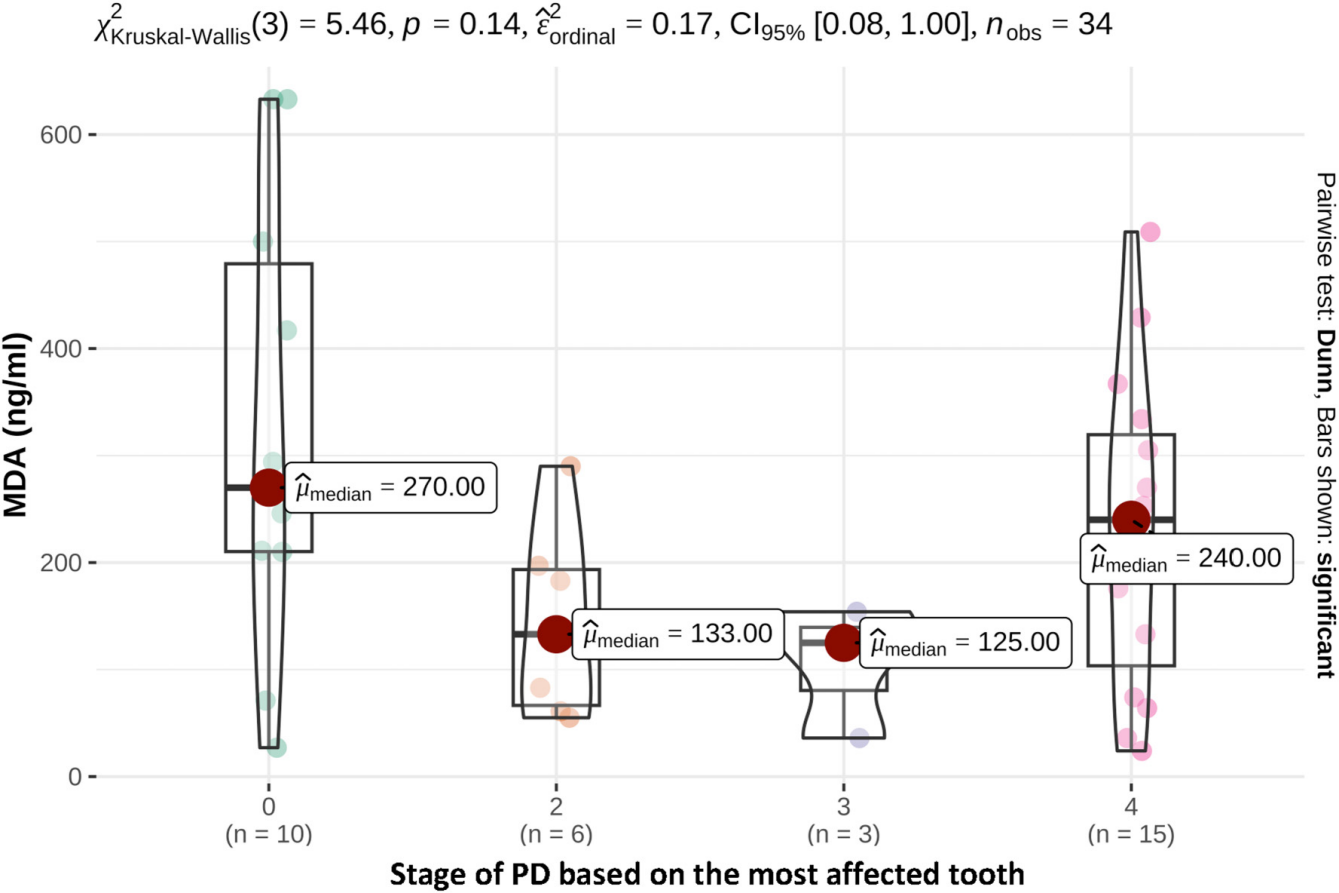
Saliva MDA concentrations (ng/ml) depending on the stage of periodontal disease of the most affected tooth.

Abbreviation: MDA, malondialdehyde.

With regard to the stage of PD of the most affected tooth, 1 patient in the study group was assigned Stage 1 (PD1), 6 patients Stage 2 (PD2), 3 patients Stage 3 (PD3) and 15 patients Stage 4 (PD4). The average MDA concentration in saliva was 133 ng/ml for patients with Stage 2 (PD 2), 125 ng/ml for Stage 3 (PD 3) and 240 ng/ml for Stage 4 (PD 4). Dogs from the control group had higher or equally high saliva MDA concentrations as patients with PD (Figure 2), although the differences were not statistically significant.

**Figure 3.**
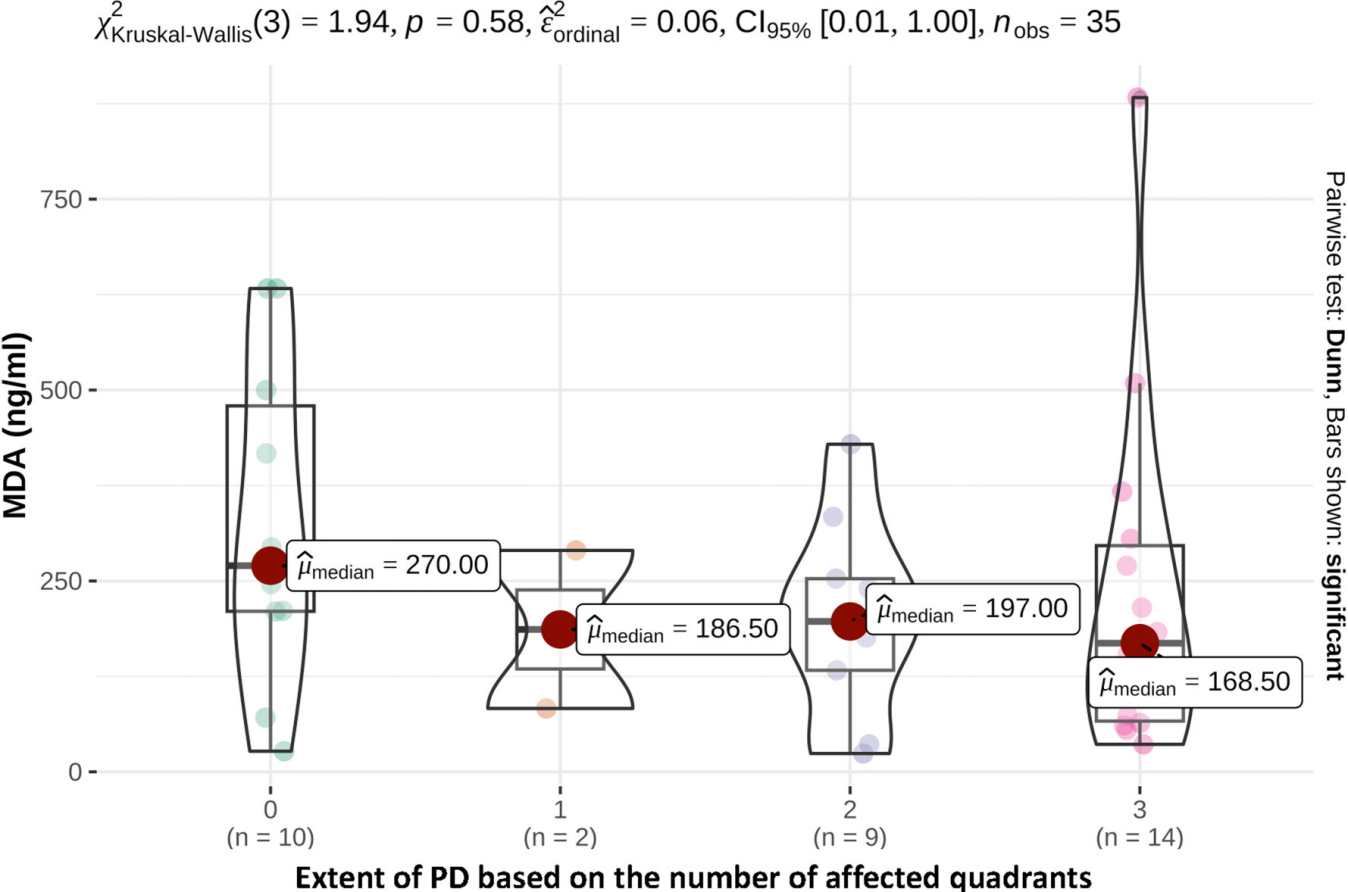
Saliva MDA concentrations (ng/ml) depending on the extent of periodontal disease based on the number of affected quadrants (table 2).

Abbreviation: MDA, malondialdehyde.

In terms of the extent of PD, 2 patients with only 1 affected tooth had a saliva MDA concentration of 185.5 ng/ml, 9 patients with several affected teeth in 1 or 2 quadrants had a saliva MDA concentration of 197 ng/ml and 14 patients with several teeth in 3 or 4 affected quadrants had a saliva MDA concentration of 168.5 ng/ml (Figure 3).

## Discussion

The present study was designed to investigate saliva MDA concentrations in dogs. The aim was to determine whether saliva MDA concentrations could be used to distinguish between dogs with and without PD. Although MDA was measured in the saliva of all dogs, no statistically significant differences were seen between affected and healthy dogs. The fact that MDA could be measured at all in the saliva of all dogs can serve as basic knowledge for further studies with dog saliva.

Whether MDA is useful as a biomarker for oxidative stress in humans with PD is controversial in the literature. In a human clinical study, 30 patients examined with chronic periodontitis and 25 patients with gingivitis, were compared to the healthy control group, which reported the patients who suffered from periodontitis had elevated levels of MDA in their saliva compared to the patients with gingivitis and the control group.^24^ In another study in human medicine on chronic periodontitis, it was shown that there was a correlation between saliva MDA concentrations and alveolar bone loss as a result of chronic periodontitis.^30^ These results are in contrast to the present study in dogs. Differences in the results between human and veterinary medicine may be related to different compositions of saliva.

If the metabolome of the dog is compared with that of humans, it becomes apparent that 25 lipids could be found in the dog that are not present in human saliva.^31^ Such differences in salivary constituents may also explain different levels of degradation products, and thus the conflicting study results. In a further study in which dog and human saliva were examined by proteome analysis, the authors were able to show that proteins related to apoptosis were found primarily in dog saliva.^32^ This could possibly explain that even physiologically in healthy dogs comparatively more degradation products of lipid peroxidation are present in saliva and the parameter may therefore not be sensitive enough to detect PDs.

However, another study in human medicine showed that MDA was not higher in saliva but only in gingival crevicular fluid in periodontitis patients compared to healthy individuals.^33^ The gingival crevicular fluid was not investigated in the present study and could possibly be analyzed in follow-up studies.

The grading of PD in the present study was based on an established staging methodology by the AVDC.^29^ However, this staging system only refers to the affected tooth. As the present study was a pilot study, the authors used both the AVDC staging and point award systems for the extent of PD-affected teeth and quadrants. As a grading system that includes both how severely a tooth is affected and how many teeth are affected does not yet exist in the accessible literature, this would be helpful for larger follow-up studies.

MDA was determined in the present study with a commercial ELISA kit, which is valid for different species and the test material. Nevertheless, the measurement of MDA in saliva has not yet been used in clinical studies in dogs. The ELISA method is a test that is already established in the literature for the determination of MDA in saliva in human medicine, not only for the detection of oxidative stress in the blood,^34^ but also in saliva in people with periodontitis.^35^

Quantifying MDA in the blood by liquid chromatography-mass spectrometry could provide more precision in a follow-up study. However, this would first have to be established for the specific animal species.

In addition to MDA, other biomarkers such as total oxidant status, glutathione peroxidase (GPx) or superoxide dismutase concentrations can be measured to detect oxidative stress in the saliva of patients with PD.^35^

It is important to bear in mind that such biomarkers are only markers of oxidative stress and, like MDA, are not specific for PD. If patients suffer from other diseases that also lead to oxidative stress, this could also influence the composition of saliva. Thus, MDA, as a biomarker for oxidative stress, is also discussed in human medicine with other diseases such as diabetes,^36^ atherosclerosis,^37^ and tumor diseases.^38^ To date no clinical studies have been conducted in dogs. Therefore, the prerequisite for participation in the present study was that the dogs in the study did not have any other reported diseases. In addition to the preliminary report of the patient, the blood count values had to be unremarkable. In the present study, such comorbidities of the dogs were unlikely but could not be excluded with complete certainty, so that could possibly have influenced the results. Surprisingly, some of the dogs in the control group showed higher MDA readings than the study group, but the reasons for this were not determined in the present study.

Studies have shown that there also seems to be an association between periodontitis in dogs and systemic diseases (e.g., liver and kidney diseases)^39^ and heart diseases^40^; but whether these are the cause or the consequence of PD often remains unclear. If there were reliable biomarkers for the early detection of periodontitis, these could also be investigated in the context of systemic diseases.

The current study enrolled 35 dogs. Experimental follow-up studies using a greater number of animals could further investigate saliva MDA concentrations in dentally healthy and diseased dogs, although the present results call into question further investigations in terms of cost and time. Dogs with inflammatory diseases (e.g., periapical radiographic lucency, tooth fracture) or tumors were excluded. This resulted in a large number of dogs subsequently not being included in the study. One difference between the study and control groups was that the controls were significantly younger than the dogs with PD. This was unavoidable as the risk of PD increases with age in dogs. Indications for anesthesia and dental radiography in the study group were frequently persistent deciduous canines or congenital malocclusions. Therefore, the control patients tended to be younger than those in the study group.

Saliva testing is becoming increasingly important in the field of laboratory diagnostics, along with the examination of blood, cerebrospinal fluid, urine and other body fluids. In cats with gingivostomatitis^41^ or PD in general,^26^ increased immunoglobulin concentrations could be measured in serum and saliva by ELISA. In addition to the determination of hormones such as cortisol, oxytocin, or vasopressin,^42–44^ the diagnosis of allergies can be carried out as well.^45^ Furthermore, proteome analysis of saliva can be indicative of metabolic dysfunction in dogs,^46^ and a pilot study has already measured elevated urea and creatinine levels in dogs with chronic kidney disease.^47^ Another well-known field of application in human medicine is gene analysis.^48^ The possibilities of saliva diagnostics thus appear manifold and should be further explored in veterinary medicine.

The investigation of other biomarkers in saliva may offer the possibility of diagnosis or early detection of PD in the saliva of dogs. These studies have not yet been conducted in dogs and could be investigated in clinical trials in the future. The main problem of advanced PD is that it is often irreversible and in the worst case, the affected tooth has to be extracted. Currently, periodontal probing and intraoral dental radiography remain the “gold standard” for diagnosing PD, which requires general anesthesia in the dog. Nonetheless, early diagnosis would be of great advantage in detecting PD and complementing the current methods of detection.
